# Nanozymes based on functionalized iridium oxide-modified gold nanoparticles for combination therapy

**DOI:** 10.1039/d5ra07054f

**Published:** 2026-02-18

**Authors:** Yanfang Hu, Kaizheng Jia, Zhijie Guo, Yige Guo, Wenshuo Hou, Xiaofei Chen, Abdukader Abdukayum

**Affiliations:** a Xinjiang Key Laboratory of Novel Functional Materials Chemistry, College of Chemistry and Environmental Sciences, Kashi University Kashi 844000 PR China abdukadera@sina.com; b School of Materials Science and Engineering, Beijing Institute of Technology Beijing 100081 PR China

## Abstract

Combination therapy has become the trend in cancer treatments. Iridium oxide-based nanoparticles have shown potential for application in tumour therapy due to their excellent photophysical-chemical and hydrogen peroxide-like enzymatic activities. However, during the practical application of functionalized iridium oxide, it was observed that a high-power near-infrared laser (NIR) is required to achieve better results. The excellent photothermal conversion efficiency of gold nanoparticles has been widely verified. Based on these facts, nanozymes based on functionalized iridium oxide-modified gold nanoparticles (PIrS@Au) were developed. PIrS@Au with good photothermal conversion efficiency, photochemical activity, catalase-like activity and biocompatibility could be effectively taken up by HeLa and HepG2 cells. *In vitro*, the growth of HeLa and HepG2 cells cultured with PIrS@Au in an oxidative environment was inhibited after irradiation with 808 nm near-infrared light for 5 minutes (2.2 W cm^−2^) twice. In particular, in the experimental group, where the concentration of PIrS@Au was the highest at 400 µg mL^−1^, the survival rates of HeLa and HepG2 cells were 35.6% and 46.8%, respectively. In conclusion, PIrS@Au shows potential application in tumour combination therapy.

## Abstract 2

A facile strategy to prepare nanozymes based on functionalized iridium oxide-modified gold nanorod is presented.

## Graphical abstract

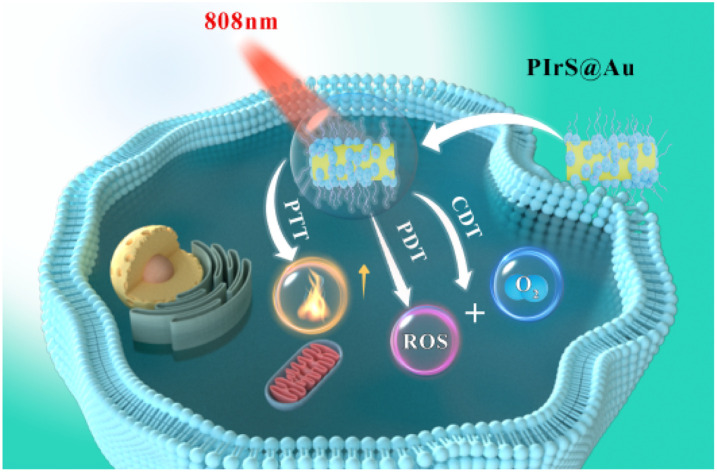

## Introduction

Nanomaterial-based artificial enzymes (nanozymes), which combine the catalytic properties of natural enzymes with those of traditional chemical catalysts, offer significant advantages such as lower cost, enhanced stability, and superior durability compared to their natural counterparts.^1–3^ Since the discovery of Fe_3_O_4_ nanoparticles as peroxidase mimics in 2007, nanozymes have garnered increasing research interest and demonstrated broad applicability in diverse fields, including biosensing, antibacterial treatments, disease diagnosis, and tumor therapy.^4–10^

Recently, iridium-based nanomaterials, with their exceptional reactive oxygen species (ROS) scavenging capacities and outstanding catalytic activities, have been widely used in biological detections, tumor therapies, and other fields.^11–14^ As early as 2020, the size effect of Pd–Ir core–shell nanoparticles with peroxidase-like activity as a model system for biosensing applications was systematically investigated using an enzyme-linked immunosorbent assay (ELISA).^15^ A new class of nanozymes (FeSA-Ir@PF NSs), constructed by conjugating supramolecular-wrapped Fe single atoms onto iridium metallene featuring lattice-strained nanoislands, enabled light-controlled production of reactive oxygen species (ROS) in terms of both quantity and variety, and they demonstrated ultrahigh photothermal conversion efficiency, cooperative robust ·OH generation, and photocatalytic O_2_/^1^O_2_ generation under NIR-II light, in addition to effective Fenton-like catalysis in the dark.^16^ Xiue Jiang and coworkers prepared bovine serum albumin–IrO_2_ nanoparticles (BSA-IrO_2_ NPs) with extraordinary photothermal (PTT) conversion efficiency and catalase-like activity.^17^ Despite these achievements, the facile functionalization of iridium-based nanomaterials remains a challenge. A commonly used functionalization strategy is to introduce macromolecules. However, most biocompatible macromolecules are photo-physically/chemically inert, which means that high-power excitation light sources are usually required in practical applications.

Gold (Au) nanoparticles have drawn widespread attention owing to their intriguing photo-physical and photochemical properties and highly promising applications in biomedical technologies.^18–22^ Numerous studies have extensively explored the use of diverse gold nanoparticle morphologies, such as nanorods, nanostars, nanospheres, nanocages, and nanosheets, for cancer treatment applications, especially in the field of PTT.^23–25^ An Au nanorod-photosensitizer complex (GNR-AlPcS4) was fabricated for noninvasive near-infrared fluorescence imaging and photo-dynamic (PDT)/PTT applications in various cancers.^26^ An innovative multifunctional theranostic nanoplatform was developed to achieve synergistic chemo-PTT-PDT, consisting of anisotropic Au nanoroses dual-loaded with doxorubicin and NIR-responsive indocyanine green.^27^ A facile strategy to construct Au nanosheets (AuNSs) functionalized with RGD peptides and small interfering RNA (siRNA) was developed to achieve NIR photoacoustic imaging-guided PTT/gene therapy.^28^

**Scheme 1:**
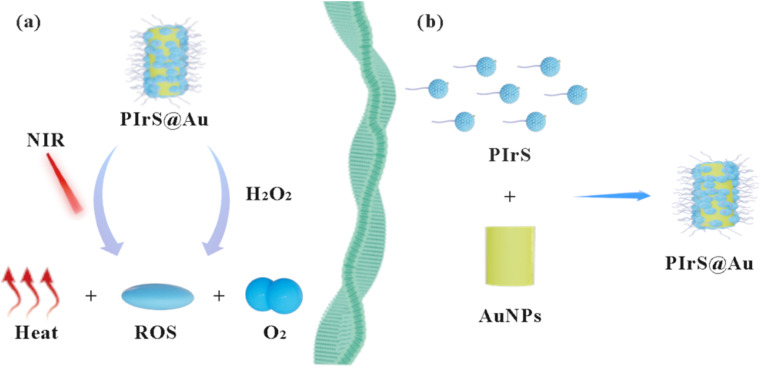
Schematic of (a) the therapeutic mechanism and (b) the synthesis route of PIrS@Au.

In this study, new nanoenzymes, functionalized iridium oxide-modified Au nanoparticles (PIrS@Au), conjugating PEGylated functional IrO_2_ (PIrS) with Au nanoparticles (AuNPs) through the gold-sulfur reaction, were developed for combination therapy. The therapeutic mechanism is shown in Scheme 1(a). The intracellular and *in vitro* anticancer efficacy indicated that PIrS@Au exhibited excellent combination therapy. This work may open another door to the construction of nanoenzymes based on functionalized iridium oxide, outlining significant potential for future biomedical applications of iridium-based nanomaterials.

## Experimental

### Materials, characterizations and preparation

Information on materials, characterizations, and preparation is shown in the SI.

### Cellular uptake and viability assays

To observe the *in vitro* uptake of PIrS@Au against HeLa and HepG2 cells, confocal laser scanning microscopy (CLSM) was employed; the procedures have been reported before and are shown in the SI.

The cytotoxic effects of PIrS@Au nanoparticles were assessed against L929 and NIH3T3 cells using a CCK-8 assay; their cytotoxicity under varying conditions was also tested on HeLa and HepG2 cells.^29^ Experimental procedures are presented in the SI in detail. Cell viability (CV) was evaluated according to eqn (1):

1 CV(%) = [(*A*_s_ − *A*_b_)/(*A*_c_ − *A*_b_)] × 100%

In eqn (1), *A*_s_, *A*_c_ and *A*_b_ represent the absorbance of groups treated with samples, control groups and blank groups, respectively.^30^

## Results and discussion

The designed nanoparticles were prepared according to the route illustrated in Scheme 1(b). AuNPs were synthesized using the seeded growth method, and hexadecyltrimethyl ammonium bromide (CTAB) acted as the template.^31^ The IrO_2_ particles were fabricated by the direct thermal hydrolysis method of IrCl_3_ under alkaline environments at 80 °C for only 10 min.^32^ Then, the amino groups were attached to the surface of IrO_2_ (IrO_2_–NH_2_). PEG with a terminal carboxyl group (mPEG-COOH, ^1^H NMR spectrum is shown in Fig. S1) and lipoic acid (LA) were grafted onto the surface of IrO_2_–NH_2_ by the classical condensation reaction to obtain the PIrS sample (Scheme S1). Finally, PIrS@Au nanoparticles were obtained by simply mixing AuNPs and PIrS with the aid of gold-sulfur bonds.

The Fourier transform infrared (FT-IR) spectra of mPEG-COOH, PIrS and PIrS@Au are shown in Fig. S2. There were peaks at 2875, 1730, 1616, 1465 and 1112 cm^−1^ in the FT-IR spectrum of mPEG-COOH, corresponding to the stretching vibration of C–H and carbonyl, antisymmetric stretching vibrations of COO^−^, bending vibration of C–H and stretching vibration of C–O–C, respectively.^33^ This spectral profile was consistent with the successful introduction of a carboxylic acid terminus to the PEG chain. The FT-IR spectrum of PIrS showed characteristic Si–C vibration peaks at 842 and 775 cm^−1^, providing direct evidence for the successful synthesis of the PEGylated hybrid structure. However, no distinct new peaks attributable to the Au–S bonds were observed in the FT-IR spectrum of PIrS@Au compared to that of PIrS. This is likely because the characteristic Au–S stretching vibration is both very weak in intensity and typically occurs at wavenumbers below 400 cm^−1^, making it difficult to detect with conventional FT-IR.

The ultraviolet-visible (UV-vis) spectra of AuNPs, PIrS, and PIrS@Au are presented in Fig. S3. The UV-vis spectrum of PIrS exhibited no significant absorption peaks across the wavelength range of 400 to 850 nm. Two absorption peaks were observed in the AuNPs spectrum, attributed to transverse (TSPR) and longitudinal (LSPR) plasmon oscillations at 537 nm and 710 nm, respectively.^34^ Following combination with PIRS, these two peaks were red-shifted to 574 and 752 nm, respectively.

The fluorescence spectra of AuNPs, PIrS, and PIrS@Au at an excitation wavelength of 405 nm are presented in Fig. S4(a). The CIE color coordinates of PIrS@Au were measured as (*x*, *y*) = (0.2276, 0.5478), indicating a pure green emission (Fig. S4(b)).

The phase and crystalline structure of AuNPs, IrO_2_, PIrS, and PIrS@Au were confirmed by X-ray diffraction (XRD). As shown in Fig. S5, the XRD pattern exhibited distinct diffraction peaks at 2*θ* values of approximately 38.30°, 44.49°, 64.69°, and 77.70°, which could be indexed to the (111), (200), (220), and (311) crystal planes, respectively, of the face-centered cubic (fcc) gold (PDF#04-004-4643(RDB)).^35^ The XRD patterns of both IrO_2_ and PIrS were very similar, showing a broad hump without sharp diffraction peaks, which indicated that both materials possess an amorphous nature. After bonding with AuNPs *via* the gold–sulfur bond, the characteristic peak attributed to IrO_2_ at 36.69° in the XRD spectrum of PIrS@Au exhibited increased numerical values relative to standard data, accompanied by a significant enhancement in intensity. However, the observed peaks at 2*θ* values of approximately 38.20° and 44.38° were smaller than those indexed to the (111) and (200) crystal planes of AuNPs, implying a hybrid structure.

**Fig. 1:**
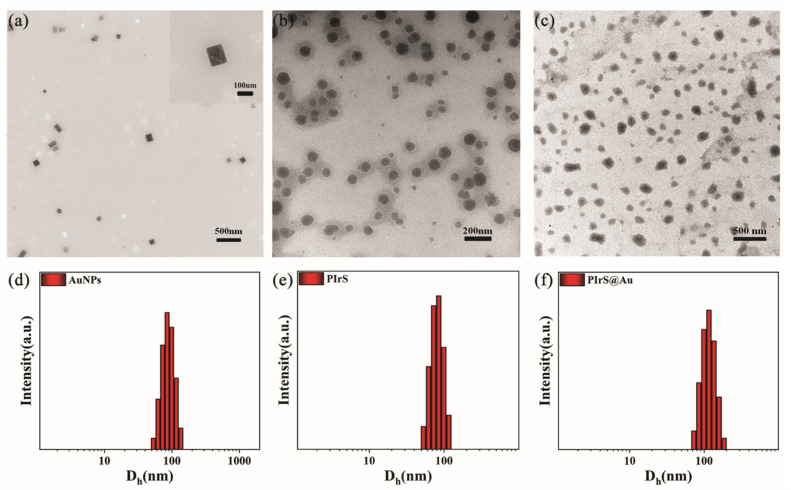
TEM images of AuNPs (a), PIrS (b) and PIrS@Au (c), and DLS of AuNPs (d), PIrS (e) and PIrS@Au (f) in an aqueous solution (100 µg mL^−1^).

The morphology and size of AuNPs, PIrS and PIrS@Au were characterized by transmission electron microscopy (TEM), along with dynamic light scattering (DLS). As presented in Fig. 1(a), TEM imaging of AuNPs revealed an average length of 92.55 ± 18.44 nm and an average width of 72.95 ± 15.47 nm, as determined by ImageJ analysis of 21 particles, with aspect ratios ranging from 1.07 to 1.34. An enlarged image of a single nanostructure is provided in the upper right corner. Further characterization by dynamic light scattering (DLS) yielded a hydrodynamic diameter (*D*_h_) of 89.30 ± 19.36 nm for the AuNPs, as presented in Fig. 1(d). TEM imaging of PIrS, shown in Fig. 1(b), confirmed a diameter of 69.64 ± 16.21 nm (determined by ImageJ analysis of 50 particles), which was consistent with the results of the DLS (*D*_h_ = 81.44 ± 15.90 nm), as shown in Fig. 1(e). For PIrS@Au, TEM imaging in Fig. 1(c) revealed a diameter of 102.05 ± 23.22 nm (determined by ImageJ analysis of 55 particles), showing agreement with the DLS results (*D*_h_ = 116.60 ± 25.15 nm) displayed in Fig. 1(f).

The stability of PIrS@Au was assessed in a serum protein environment (PBS containing 0.5% bovine serum albumin). As shown in Fig. S7 and Table S1, over 7 days, the DLS value showed no significant change, shifting from 116.35 ± 45.30 nm (0 days) to 132.77 ± 39.30 nm (7 days). These results indicated good colloidal stability in terms of size distribution in a serum protein environment.

X-ray photo-electron spectroscopy (XPS) was conducted to study the surface morphology of AuNPs, IrO_2_, PIrS and PIrS@Au. Compared to the XPS spectrum of IrO_2_ (Fig. s8(a)), a noticeable change in the peak intensity ratio of O 1s/Ir 4f was observed in the XPS spectra of PIrS and PIrS@Au, which could be attributed to the surface modification of IrO_2_. In the Ir 4f region of the XPS spectrum of IrO_2_ (Fig. S8(b)), there were two characteristic peaks at 64.8 and 61.8 eV assigned to 4f_5/2_ and 4f_7/2_ of Ir^4+^, however, following surface modification, the 4f_5_/_2_ peak shifted negatively to 60.6 eV, while the 4f_7_/_2_ peak split into two components at 63.9 and 63.5 eV during this negative shift.^36,37^ The Au 4f XPS spectra of AuNPs (Fig. S8(c)) exhibited the characteristic doublet of metallic gold (Au^0^) at 84.0 eV (4f_7_/_2_) and 87.6 eV (4f_5_/_2_).^38^ In contrast, significant changes were observed in the spectrum of PIrS@Au. The 4f_7_/_2_ peak shifts positively to 84.2 eV while simultaneously forming a new peak with a value of 84.6 eV, and the 4f_5/2_ peak broadened, which collectively implied the formation of the gold-sulphur bonds.^39^

The zeta potential values for aqueous solutions of AuNPs, IrO_2_, IrO_2_–NH_2_ PIrS and PIrS@Au are shown in Fig. S9. Surface functionalization with APTES reversed the surface charge of IrO_2_ from −32.7 mV to +12.6 mV, confirmed by zeta potential measurements. However, the PIrS surface exhibited negative charge (zeta potential: −13.8 mV) due to PEGylation. After combining with positively charged AuNPs (+11.65 mV), the zeta value of PIrs@Au increased slightly compared to that of PIrS, but ultimately remained negative at −9.77 mV.

**Fig. 2:**
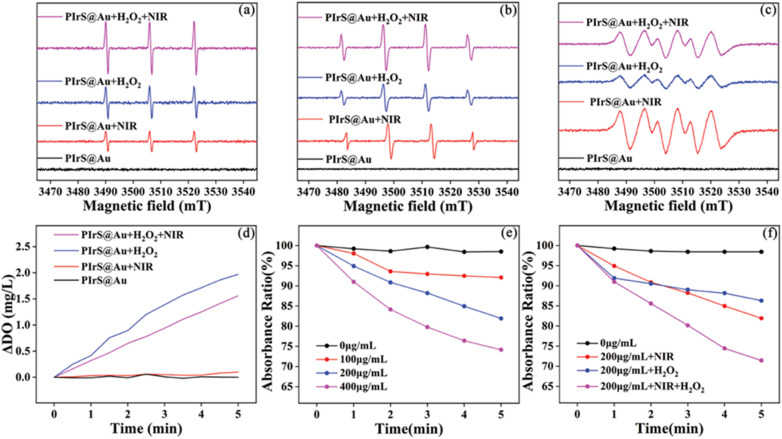
DMPO-^1^O_s_ (a), DMPO-^·^OH (b) and DMPO-^·^O_2_^−^ (c) EPR spectra of PIrs@Au (black), PIrs@Au irradiated by NIR for 5 minutes (red), PIrs@Au with 10 mM H_2_O_2_ (blue), PIrs@Au with 10 mM H_2_O_2_ irradiated by NIR for 5 minutes (red). (d) Variation of DO content in an aqueous solution of PIrS@Au (black), PIrS@Au + NIR (red), PIrS@Au + H_2_O_2_ (blue), and PIrS@Au + H_2_O_2_ + NIR (pink). Time-dependent absorbance change of DPBF (*A*/*A*_0_) at 412 nm with (e) different concentrations of PIrS@Au (0, 100, 200 and 400 µg mL^−1^) under NIR irradiation, and (f) 200 µg mL^−1^ of PIrS@Au in different conditions.

Electron spin resonance (ESR) spectra were employed to detect ROS generated by NIR irradiation (808 nm) in a normal or hydrogen peroxide (H_2_O_2_)-rich environment. As shown in Fig. 2(a)–(c), the signals of 5,5-dimethyl-1-pyrroline *N*-oxide (DMPO)-^1^O_s_/–^·^OH/–^·^O_2_^−^ adducts were observed after PIrs@Au were irradiated by NIR for 5 min; however, without the irradiation, no signal was observed in the groups of PIrs@Au. After H_2_O_2_ (10 mM) was added to the aqueous solution of PIrs@Au for 5 min, the signals of DMPO-^1^O_s_/–^·^OH/–^·^O_2_^−^ adducts were captured. Compared to normal environments, the signals of DMPO-^1^Os and –^·^OH adducts were strengthened after irradiation by NIR for 5 min in a H_2_O_2_-rich environment. However, after irradiation with NIR for 5 min in a H_2_O_2_-rich environment, the slight reduction of signals in the DMPO-^·^O_2_^−^ adducts was noticed.

To test the catalase (CAT)-like activity, real-time monitoring of dissolved oxygen (DO) levels was performed on a 10 mL PIrS@Au aqueous solution (200 µg per mL) under different conditions with constant stirring using a portable dissolved oxygen meter, automated measurements were recorded at 30-seconds intervals over a 5-minutes observation period (Fig. 2(d)). Additionally, the same amount of water was given the same treatment as the control (Fig. S10). The DO was hardly detected in water (black), water (red), water with H_2_O_2_ (10 mM, blue), and water with H_2_O_2_ (10 mM) under NIR (pink). In the PIrS@Au aqueous solution, DO was hardly detected; while PIrS@Au nanoparticles were placed in an oxidative environment, the DO was obviously detected. However, it is notable that the produced O_2_ by PIrS@Au/H_2_O_2_ was consumed after NIR treatment, which suggests some kind of transformation.

To test the photo-/chemo-dynamic ability of PIrs@Au to generate ROS under NIR irradiation, 1,3-diphenylisobenzo-furan (DPBF), which could respond to ROS, was chosen. As illustrated in Fig. 2(e), the UV-vis absorption of DPBF decreased in the presence of PIrs@Au under NIR irradiation, attributed to ROS generation *via* the photocatalytic process. As the concentration of PIrs@Au increased, the photodynamic effect was significantly enhanced. These results confirm the efficient photodynamic activity of PIrs@Au under NIR excitation and suggest its potential value as a photosensitiser. As depicted in Fig. 2(f), upon adding 10 mM H_2_O_2_ to a solution of PIrs@Au (200 µg per mL), the UV-vis absorption of DPBF dropped sharply within 1 min. Under NIR irradiation, the absorption of the solution containing PIrs@Au (200 µg per mL), H_2_O_2_ (10 mM) and DPBF (10 µL, 10 mM in ethanol) continued to decrease over time. This progressive decline was attributed to the generation of ROS through synergistic photo- and chemo-catalytic processes. The markedly accelerated degradation kinetics in the presence of H_2_O_2_ highlight the potential of PIrs@Au to act as an efficient photosensitiser and catalyst for enhanced ROS production *via* combined photodynamic and chemodynamic pathways, which may broaden its applicability in ROS-mediated therapies.

**Fig. 3:**
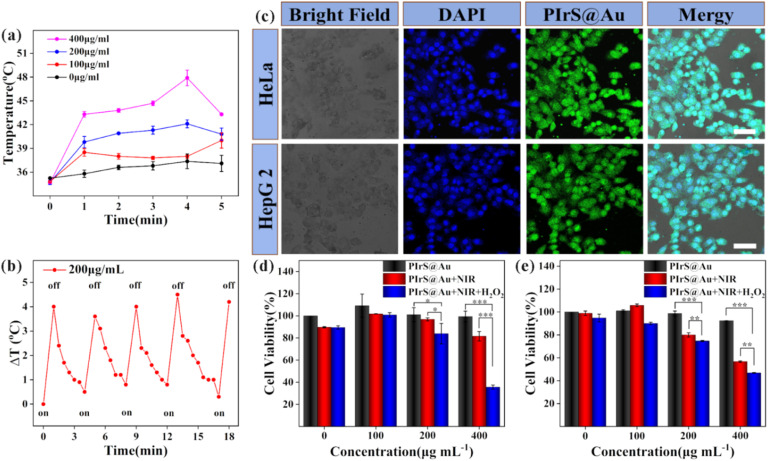
(a) Photothermal effect of aqueous solutions of PIrS@Au with different concentrations (100, 200, and 400 µg per mL) under the irradiation of 808-nm laser (2.2 W cm^−2^) for different time periods. (b) Temperature curve of aqueous solutions of PIrS@Au (200 µg per mL) during five cycles of continuous laser irradiation. (c) Cellular uptake of PIrS@Au after incubation with HeLa and HepG2 cells for 2 hours. The scale bars are 50 µm. Cell viability of HeLa (d) and HepG2 cells (e) after 24 h treatment with PIrS@Au (black), PIrS@Au + NIR irradiation (red), and PIrS@Au + H_2_O_2_ (1 mM) + NIR irradiation (blue). Error bars represent the experimental range of triplicate measurements; *p < 0.05, **p < 0.01, and ***p < 0.001.

To evaluate the photothermal effect driven by NIR irradiation, different concentrations of aqueous solutions of PIrS@Au (100, 200 and 400 µg per mL) were irradiated with NIR laser for 5 minutes, as shown in Fig. 3(a). After being irradiated with NIR, for samples with 200 and 400 µg mL^−1^ of PIrS@Au, the temperature of the solution rose to the highest temperature (42.1 and 47.9 °C, respectively) within 4 minutes. Even with the lowest concentration (100 µg mL^−1^), the temperature could rise by 3.7 °C in the first minute. Moreover, the photothermal stability of PIrS@Au was verified in successive on/off cycle tests of NIR irradiation (Fig. 3(b)). The results indicated that the designed samples, PIrS@Au with excellent photothermal performance, could be used for PTT.

Confocal Iaser scanning microscopy (CLSM) was employed to observe the *in vitro* cellular uptake of PIrS@Au against HeLa and HepG2 cells. As demonstrated in Fig. 3(c), the HeLa and HepG2 cells incubated with PIrS@Au exhibited significantly strong intracellular fluorescence in the entire cell, indicating that PIrS@Au could be effectively taken up by HeLa and HepG2 cells.

The cytotoxicity of PIrS@Au was first evaluated. CCK-8 assay results (Fig. S11) demonstrated that PIrS@Au exhibited minimal cytotoxicity toward L929 and NIH3T3 cells. No significant cell death was observed, and the cell survival rate remained above 95% even at 400 µg mL^−1^, further confirming its high biocompatibility.

The cytotoxicities of PIrS@Au, PIrS@Au with NIR irradiation, and PIrS@Au with NIR irradiation in an oxidative environment were evaluated using CCK-8 assays. CCK-8 assay results (Fig. 3(d) and (e)) demonstrated that PIrS@Au exhibited minimal cytotoxicity toward HeLa and HepG2 cells, with no significant cell death observed. Control-group experiments confirmed that NIR irradiation alone had a negligible impact on cell viability under either normal physiological or oxidative stress conditions. However, after being irradiated with NIR for 5 minutes twice, the growth of HeLa and HepG2 cells cultured with PIrS@Au in both normal and oxidative environments was inhibited. In particular, in the experimental group where the concentration of PIrS@Au reached the highest at 400 µg mL^−1^, the survival rates of HeLa and HepG2 cells in the oxidative environment were 35.6% and 46.8%, respectively. These results demonstrate that PIrS@Au has potential application in tumour combination therapy.

## Conclusions

In conclusion, a facile strategy to synthesize nanoenzymes based on functionalized iridium oxide-modified gold nanoparticles (PIrS@Au) for combination therapy was presented. Firstly, AuNPs were synthesized using the seeded growth method. The IrO_2_ particles were fabricated by the direct thermal hydrolysis method, and amino groups were then attached to the surface of IrO_2_. After the surface modification of iridium oxide with amino groups through the classical condensation reaction was completed, the designed nanoenzymes, PIrS@Au, were achieved by simply mixing with the aid of gold-sulfur bonds. The results demonstrated that PIrS@Au with good photothermal conversion efficiency, photochemical activity, enzyme-like activity and biocompatibility, could be effectively taken up by HeLa and HepG2 cells. *In vitro,* the growth of HeLa and HepG2 cells cultured with PIrS@Au in the oxidative environment were inhibited after irradiation with NIR for 5 minutes twice. In conclusion, PIrS@Au has potential application in tumour combination therapy.

## Author contributions

Yanfang Hu: conceptualization, data curation, funding acquisition, and writing – original draft. Zhejie Guo: writing – original draft. Kaizheng Jia, Guo Yige, and Wenshuo Hou: data curation. Xiaofei Chen: writing review and editing. Abdukader Abdukayum: writing review and editing, supervision and funding acquisition.

## Conflicts of interest

There are no conflicts to declare.

## Supplementary Material

RA-016-D5RA07054F-s001

## Data Availability

Data for this article, including supplementary information (SI), are available. Supplementary information is available. See DOI: https://doi.org/10.1039/d5ra07054f.
