# Supplementary material for: Nanozymes based on functionalized iridium oxide-modified gold nanoparticles for combination therapy
Source: RSC Adv. 2026 Feb 18;16(11):9659–65. doi: 10.1039/d5ra07054f (PMC12914648; doi:10.1039/d5ra07054f)
Supplement: RA-016-D5RA07054F-s001 [file RA-016-D5RA07054F-s001.pdf]

Supplementary Information (SI) for RSC Advances.
This journal is © The Royal Society of Chemistry 2026

Supporting Information

Nanozymes based on functionalized iridium oxide modified gold nanoparticles for combination therapy

Yanfang Hu,[1] Kaizheng Jia,[1] Zhijie Guo,[1] Guo Yige,[1] Wenshuo Hou,[1] Xiaofei Chen[2] and Abdukader Abdukayum*[1]

[1]Xinjiang Key Laboratory of Novel Functional Materials Chemistry, College of Chemistry and Environmental Sciences, Kashi University, Kashi, 844000, PR China.

[2]School of Materials Science and Engineering, Beijing institute of Technology, Beijing, 100081, PR China.

**Corresponding authors: Prof. Dr. A. Abdukayum (E-mail: abdukadera@sina.com)*

Contents

Materials 1
Characterizations...1
Synthesis of Au nanorods (AuNRs)..2
Synthesis of mPEG-COOH...2
Synthesis of IrO_2 2
Surface modification of IrO_2...3
Synthesis of PEGylated functional IrO_2 (PIrS)..3
Synthesis of functionalized iridium oxide-modified Au nanorods (PIrS@Au)..........................3
Oxygen Generation 3
Extracellular Reactive Oxygen Species (ROS) Detection 4
Confocal laser scanning microscopy (CLSM) 4
Cytotoxicity of PIrS@Au...4
Photo-therapy analyses 5
Combination therapy analyses 5
Figure S1 ^{1}H NMR spectrum of mPEG-COOH. 6
Scheme S1 Synthesis route of PIrS..6
Figure S2 FT-IR spectra of mPEG-COOH, PIrS and PIrS@Au. 6
Figure S3 Uv-vis spectra of AuNPs, PIrS and PIrS@Au. 6
Figure S4 (a) Fluorescence spectra (λ_{ex}=405 nm) of AuNPs, PIrS and PIrS@Au and (b) CIE of PIrS@Au...7
Figure S5 X-ray diffraction (XRD) patterns of IrO_2, PIrS, PIrS@Au and AuNPs.....................7
Figure S6. High-resolution TEM image of Au nanoparticles..8
Figure S7 The hydrodynamic diameter (D_h) of PIrS@Au in water solution (0.2 mg mL^{-1}) measured over different days. 8
Table S1 The D_h values of PIrS@Au in water solution (0.2 mg mL^{-1}) measured over different days. 8
Figure S8 (a) XPS survey spectra of AuNPs, IrO_2, PIrS and PIrS@Au. (b) Selective XPS survey spectrum corresponding to Ir 4f spectra of IrO_2 and PIrS. (c) Selective XPS survey spectrum corresponding to Au 4f spectra of AuNPs and PIrS@Au. 9
Figure S9 Zeta potential of aqueous solutions of Au nanorods, IrO_2, IrO_2-NH_2, PIrS and PIrS@Au. 9
Figure S10 Variation of DO content in Water (black), Water + NIR (red), Water + H_2O_2 (blue), and Water + H_2O_2+ NIR (pink). 9
Figure S11 Cell viability of L929 and NIH3T3 cells after 24 h treatment with different concentrations of PIrS@Au. 10

Materials

Iridium trichloride ($IrCl_3$, 99.9%) was purchased from Adamas Reagent, Ltd (Shanghai, China). N-Hydroxy succinimide (NHS, 99%), Hexadecyl trimethyl ammonium bromide (CTAB, 99%), 1,3-diphenylisobenzofuran (DPBF, 97%), 1-ethyl-3-(3-dimethylaminopropyl) carbodiimide hydrochloride (EDC·HCl, 98.5%), succinic anhydride (SA), L-ascorbic acid (99%) and (3-Aminopropyl)triethoxysilane (APTES, 98%) were purchased from Macklin Reagent, Ltd (Shanghai, China). Lipoic acid (LA, 99%) was purchased from Aladdin Reagent Industrial, Ltd. (Shanghai, China). $HAuCl_4$ was purchased from AiYan Reagent, Shanghai Tengzhun Biotechnology Co., Ltd. (Shanghai, China). Poly(ethylene glycol) methyl ether (mPEG, M_n=2000) was purchased from Bidepharm Ltd (Shanghai, China). All chemicals were used as received.

HeLa cells (No. SCSP-504), HepG2 cells (No. SCSP-510), and L929 cells (No. GNM28) were provided by the Stem Cell Bank of the Chinese Academy of Sciences (Shanghai). NIH3T3 cells (No. QS-M018) were purchased from Keycell Biotechnology (Wuhan) Co., Ltd.

Characterizations

The chemical structures of PEG with terminal carboxyl group **(**mPEG-COOH**)** were characterized by ^1H NMR spectra on Bruker AV 400 NMR spectrometer in deuterodichloromethanee (CD_2Cl_2). The fourier transform infrared spectroscopy (FT-IR) spectra of the compounds were obtained from Thermo IR-200, Thermo Fisher Scientific, USA. Transmission electron microscopy (TEM) image were collected on a HITACHI HT7820 transmission electron microscope with an accelerating voltage of 100 kV. The hydrodynamic diameter of those nanoparticles (D_h, scattering angle at 90°) and zeta potential were measured by BeNano 180 Zeta Pro (Dandong bettersize Instruments Co). The infrared diode laser model was purchased from Changchun New Industries Optoelectronics Tech. Co., Ltd. (MDL-XF-808-10W), and the temperature changes of the samples exposed under NIR

during 5 mins were tracked by a UTi260A thermal imager from the UNI-TREND Technology (China) Co., Ltd. X-ray photoelectron spectra (XPS) were obtained by Thermo Fisher Scientific K-Alpha, USA. The portable dissolved oxygen meters (JPBJ-608, Shanghai instrument scientific instrument Co., LTD. Shanghai, China) was used to record the dissolved oxygen content. The UV-Vis absorption spectra were tested by UV-2600 of SHIMADZU, Japan. Photoluminescence spectra were recorded on an FS5C spectrofluorometer (Edinburgh Instruments, UK). Electron paramagnetic resonance (EPR) measurement was conducted by Bruker EMXNANO (Germany).

Synthesis of Au nanorods (AuNRs)

All processes were operated in the summer.

Firstly, the seed solution was prepared. Briefly, 21 μL of an aqueous solution of $HAuCl_4$ solution (58.85mM) was added to 5 mL of CTAB solution (0.10 M) in a round-bottom flask. Then, 300 μL of an aqueous $NaBH_4$ solution (10 mM) was added all at once, followed by rapid inversion mixing for 2 min. After that, the system was kept in a water bath maintained at 30 °C to obtain stable seed solution. The growth solution was prepared by mixing 10 mL of CTAB (0.1 mM), 85 mL of $HAuCl_4$ (58.85mM) and 190 μL of HCl (1M) together. Another 120 μL of $AgNO_3$ (10 mM) was added into the above solution. Following this step, 1 mL of L-ascorbic acid (0.1 M) was added to the resulting solution. Finally, 24 μL of seed solution was added to the resulting solution. The temperature of the growth medium was kept constant at 27~30 ºC during the whole procedure.

Synthesis of mPEG-COOH

mPEG (20 g, 10 mmol), succinic anhydride (5.0 g, 50 mmol), and DMAP (0.244 g, 2.0 mmol) were dissolved in 35 mL of dichloromethane (CH_2Cl_2). The mixture was stirred at room temperature for 48 hours. After that, the solution was filtrated and precipitated in ethyl ether twice to obtain mPEG-COOH (yield, 90.1 %).

Synthesis of IrO_2

$IrCl_3$ (75 mg) was added into 25 mL water, the mixture was stirred for 3 h and then kept in 4°C for 3~4 days until the mixture became clear. After the temperature of the $IrCl_3$ aqueous solution returned to room temperature, NaOH solution (1.0 mol • L^{-1}) was added drop-wise to adjust the pH value to 12. The mixture was placed into oil bath under vigorous stirring and reaction temperature was set to 80°C. Then, the mixture was allowed to be continued to react for 10min. The products were freeze-dried and then collected for further use.

Surface modification of IrO_2

IrO_2 (50mg) and APTES (0.287mL) were added to 100 mL ethanol and sonicated until the mixture was dispersed well. Then the mixture was stirred at 80 ℃ for 12 h. Then, the suspended substances were separated by centrifugation and dried in a vacuum oven at 50 ℃ for 10 h.

Synthesis of PEGylated functional IrO_2 (PIrS)

After stirred for 12 h, 5 ml of DMF solution which contained mPEG-COOH (110 mg), HNS (12.7 mg) and EDC • HCl (32.1mg) was added into 15ml of DMF solution containing 30mg of IrO_x-NH_2. After the mixture was stirred for 48 h in room temperature, the other 5 ml of DMF solution containing LA (11.5mg), HNS (12.7mg) and EDC·HCl (32.1mg) that had been stirred for 12 h, was added drop-wise. The whole mixture was stirred for another 48h. The sample of mPEG-IrO_2-LA was obtained by dialysis and lyophilisation.

Synthesis of functionalized iridium oxide-modified Au nanorods (PIrS@Au)

AuNRs and PIrs were mixed uniformly in 15 mL of deionized water at a mass ratio of 2: 1 (AuNRs: PIrS = 20mg: 10mg), and stirred overnight in the dark. The sample of PIrS@Au was obtained by dialysis and lyophilisation.

Oxygen Generation

Changes in the oxygen (O_2) content of 10 mL of PIrS@Au (200 μg mL^{-1}) aqueous solutions in different conditions under constant stirring for 5 minutes were recorded with a portable dissolved oxygen meter: (1) 10 mL of PIrS@Au aqueous solution under constant

stirring was recorded for 5 min; (2) 10 mL of PIrS@Au aqueous solution was irradiated by NIR for 5 min with vigorous stirring. (3) 10 mM H_2O_2 was added in 10 mL of PIrS@Au aqueous solutions, and then aqueous solution was recorded for 5 min under vigorous stirring condition; (4) 10 mL of PIrS@Au aqueous solutions with 10 mM H_2O_2 were irradiated by NIR for 5 min under vigorous stirring condition. The variation of dissolved oxygen (O_2) was automatically recorded every 30 s. 10 mL of water solution were treated in the same way as for control groups.

Extracellular Reactive Oxygen Species (ROS) Detection

DPBF was employed to evaluate the ROS generation of PIrS@Au in different conditions by UV-vis absorption spectra: (1) 10 μL of DPBF solution (10 mM in ethanol) was added into 1 mL of PIrS@Au (0, 100, 200 and 400 μg mL^{-1}) aqueous solution. Then, the mixture was irradiated with an 808 nm laser (2.2 W cm^{-2}) for 5 min; (2) 10 μL of DPBF solution (10 mM in ethanol) was added into 1 mL aqueous solution containing PIrS@Au (200μg mL^{-1}) and 10 mM H_2O_2, and then the absorbance of the mixture was recorded for 5 min; (3) 1 mL of PIrS@Au (200μg mL^{-1}) aqueous solution with H_2O_2 (10 mM) and DPBF (10 μL, 10 mM in ethanol) was irradiated with an 808 nm laser for 5 min. The absorption of DPBF at 412 nm was recorded every 1 min.

Confocal laser scanning microscopy (CLSM)

The HeLa and HepG 2 cells were separately seeded on pre-sterilized glass sheets in 6-well plates at a density of $2.0*10^5$ cells per well in 2.0 mL of complete high glucose Dulbecco's modified Eagle's medium (HGDMEM). Then those cells were incubated at 37°C for 24 h containing 5% (v/v) carbon dioxide (CO_2). Subsequently, the cells were incubated at 37°C for additional 2 h with 100 μg mL^{-1} of PIrS@Au. Afterwards, the cells on glass sheets were washed with PBS more than three times and immobilization by 4% (w/v) PBS buffered paraformaldehyde for 20 min at room temperature. The cells then were washed with PBS

three times, stained with DAPI for 5 min. The microimages of cells were obtained on a laser scanning confocal microscopy (CLSM, LSM 780, Carl Zeiss, Jena, Germany).

Cytotoxicity of PIrS@Au

The L929 and NIH3T3 cells were seeded in 96-well plates at 8.0 $\times 10^3$ cells per well in 180.0 μL of complete HG-DMEM, and then incubated at 37 °C in 5 % (v/v) CO_2 for 24 h. Subsequently, PIrS@Au nanoparticles were added respectively to wells with a concentration range from 50 to 400 mg mL^{-1}. After incubation for 24 h, a CCK-8 solution was added and the absorbance at 450 nm was determined by a Spark™ Multimode microplate reader (Tecan, Switzerland).

Photo-therapy analyses

The HeLa and HepG 2 cells were seeded in 96-well plates at 8.0 $\times 10^3$ cells per well in 180.0 mL of complete HG-DMEM, and incubated at 37 ^oC in 5% (v/v) CO_2 for 24 h. Subsequently, PIrS@Au nanoparticles were added respectively to wells with a concentration range from 0, 100, 200 and 400 mg mL^{-1}. For the laser treatment groups, the cells were irradiated by an 808 nm NIR laser for 5 min after incubation for 4 h. After another 4 h, the cells of the laser treatment groups were irradiated by an 808 nm NIR laser for another 5 min. After totally incubation 24 h, a CCK-8 solution was added and the absorbance at 450 nm was determined by a Spark™ Multimode microplate reader (Tecan, Switzerland).

Combination therapy analyses

The HeLa and HepG 2 cells were seeded in 96-well plates at 8.0 $\times 10^3$ cells per well in 180.0 mL of complete HG-DMEM, and incubated at 37 ^oC in 5% (v/v) CO_2 for 24 h. The cells were pre-incubated with 1 mM of H_2O_2 for 2 h. Subsequently, PIrS@Au nanoparticles were respectively added to wells with a concentration range from 100 to 400 mg mL^{-1}, respectively. For the laser treatment groups, the HeLa cells were irradiated by an 808 nm NIR laser for 5 min after incubation for 4 h. After another 4 h, the HeLa cells of the laser treatment groups were irradiated by an 808 nm NIR laser for another 5 min. After incubation 24 h, a

CCK-8 solution was added and the absorbance at 450 nm was determined by a Spark™ Multimode microplate reader (Tecan, Switzerland).

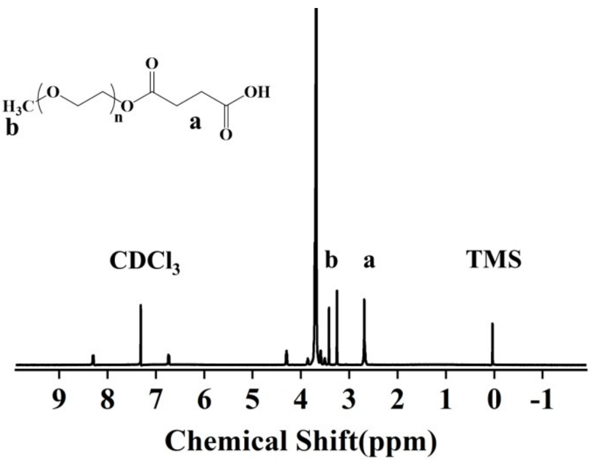

Figure S1 ^{1}H NMR spectrum of mPEG-COOH.

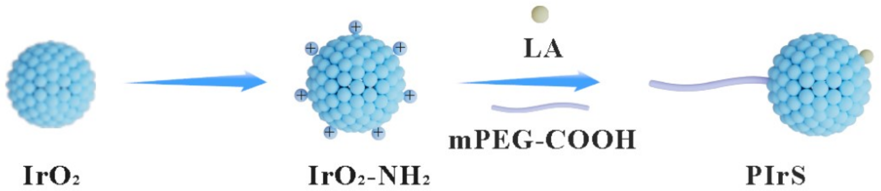

Scheme S1 Synthesis route of PIrS.

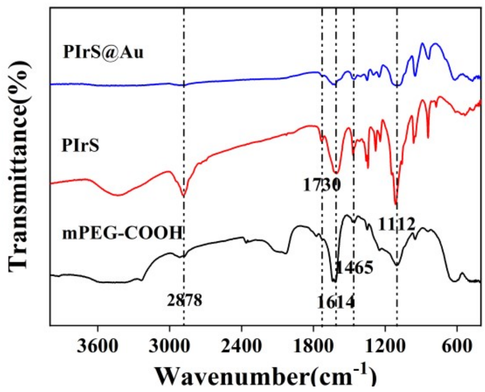

Figure S2 FT-IR spectra of mPEG-COOH, PIrS and PIrS@Au.

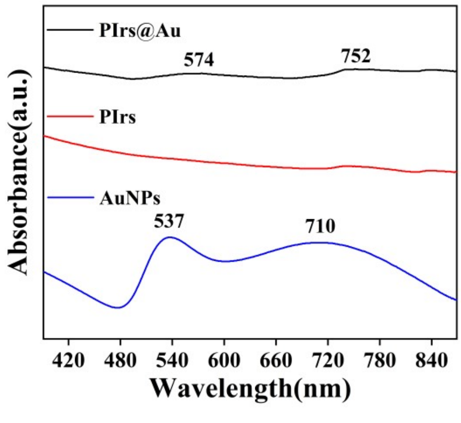

Figure S3 Uv-vis spectra of AuNPs, PIrS and PIrS@Au.

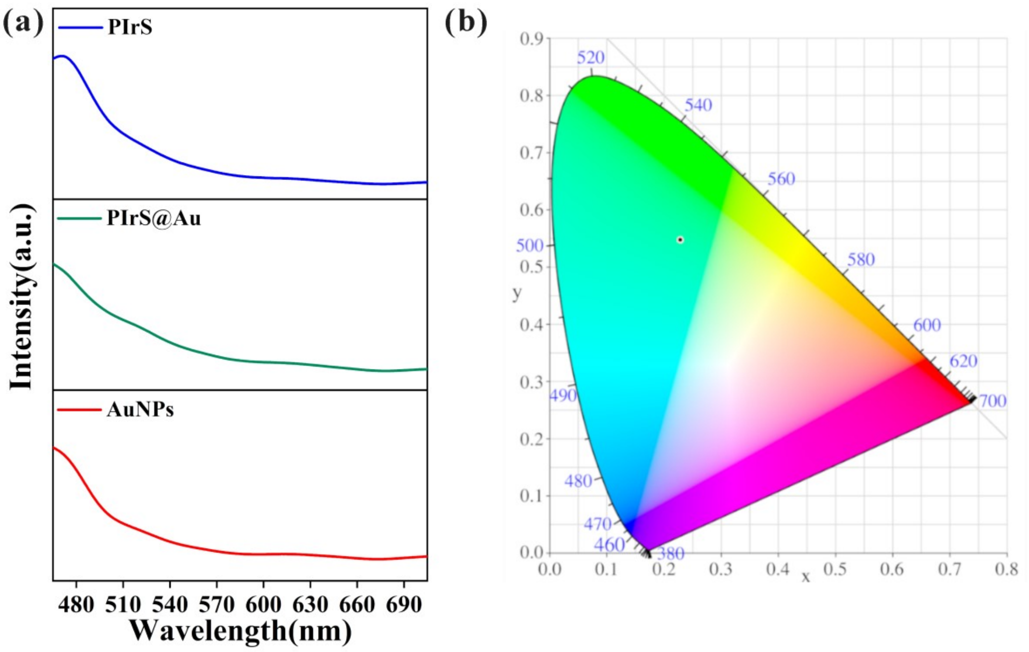

Figure S4 (a) Fluorescence spectra (λ_{ex}=405 nm) of AuNPs, PIrS and PIrS@Au and (b) CIE of PIrS@Au.

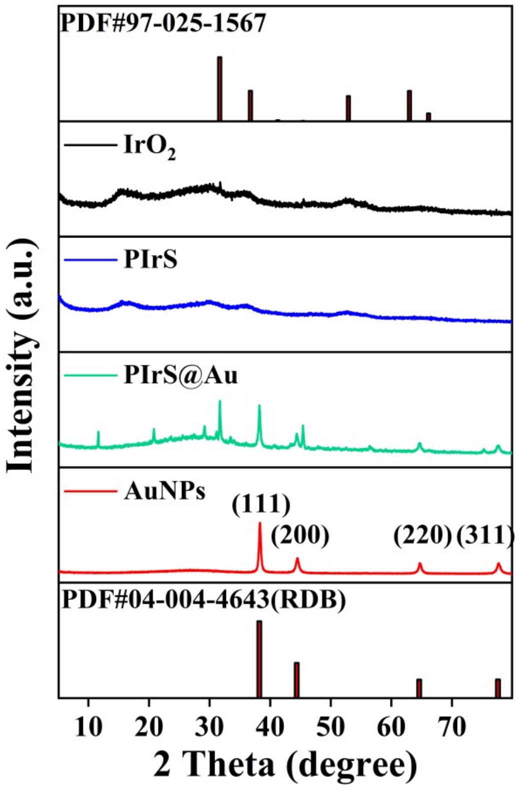

Figure S5 X-ray diffraction (XRD) patterns of IrO_2, PIrS, PIrS@Au and AuNPs.

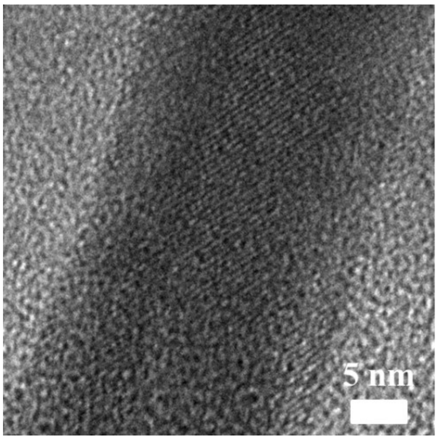

Figure S6. High-resolution TEM image of Au nanoparticles.

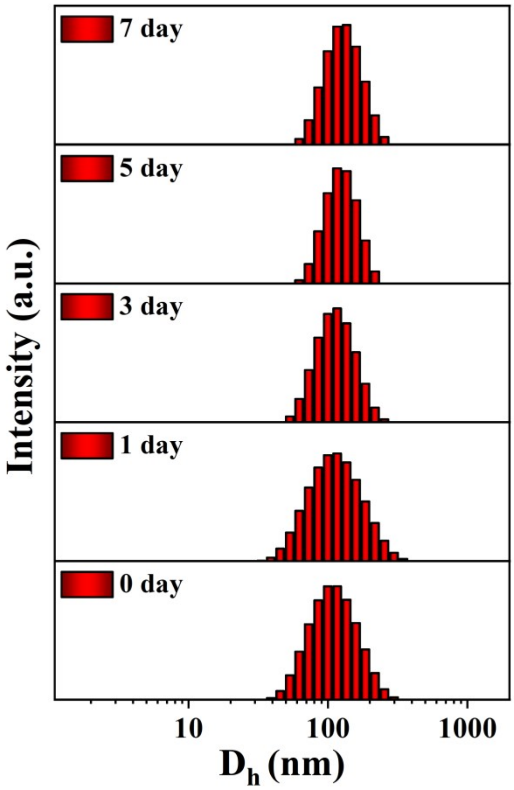

Figure S7 The hydrodynamic diameter (D_h) of PIrS@Au in water solution (0.2 mg mL^{-1}) measured over different days.

Table S1 The D_h values of PIrS@Au in water solution (0.2 mg mL^{-1}) measured over different days.

	0 day	1 day	3 day	5 day	7 day
D_h (nm)	116.35 ± 45.30	122.00 ± 53.10	117.99 ± 37.41	127.11 ± 33.26	132.77 ±39.30

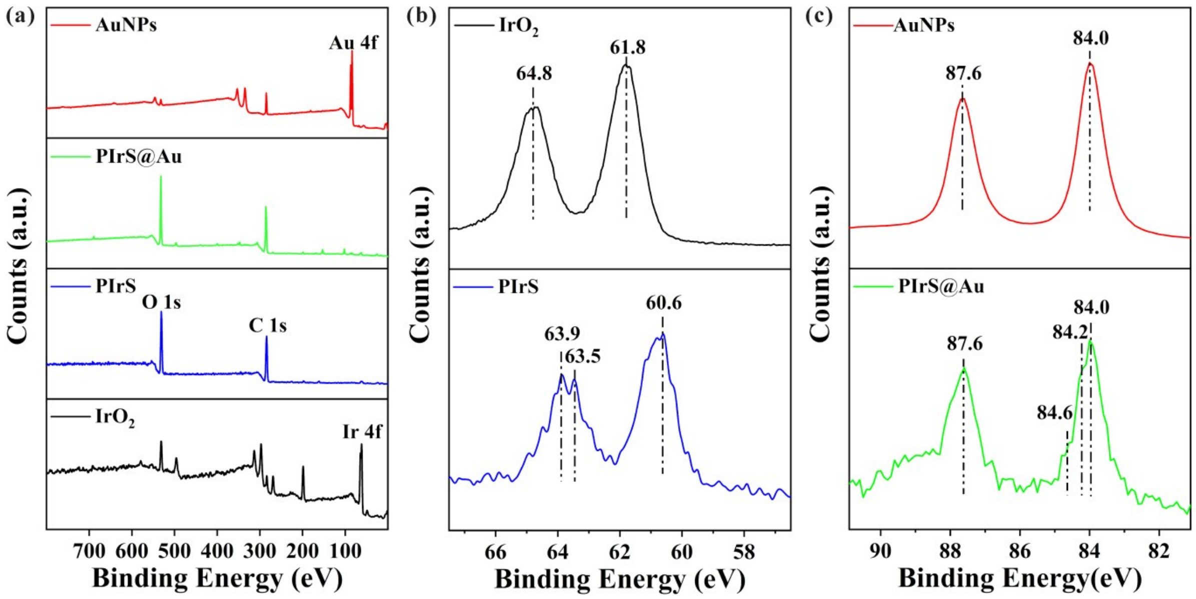

Figure S8 (a) XPS survey spectra of AuNPs, IrO_2, PIrS and PIrS@Au. (b) Selective XPS survey spectrum corresponding to Ir 4f spectra of IrO_2 and PIrS. (c) Selective XPS survey spectrum corresponding to Au 4f spectra of AuNPs and PIrS@Au.

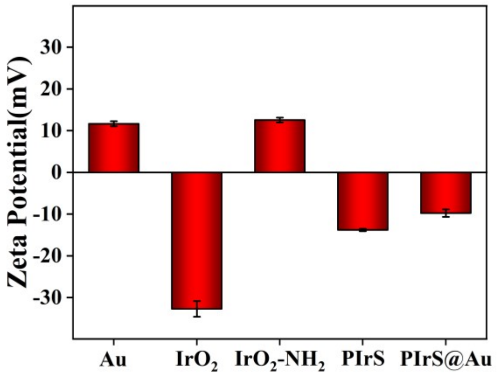

Figure S9 Zeta potential of aqueous solutions of Au nanorods, IrO_2, IrO_2-NH_2, PIrS and PIrS@Au.

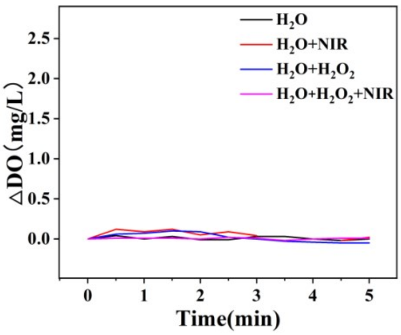

Figure S10 Variation of DO content in Water (black), Water + NIR (red), Water + H_2O_2 (blue), and Water + H_2O_2+ NIR (pink).

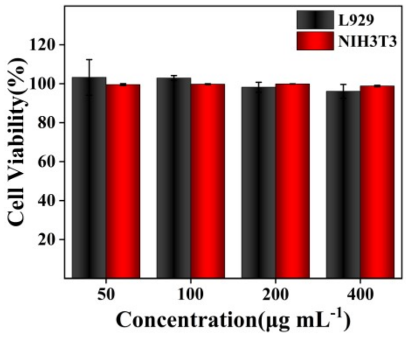

Figure S11 Cell viability of L929 and NIH3T3 cells after 24 h treatment with different concentrations of PIrS@Au.
